# Chronic obstructive pulmonary disease: in-depth analysis of microbiota association and innovative prevention and treatment approaches from the gut-lung axis perspective

**DOI:** 10.3389/fimmu.2025.1549865

**Published:** 2025-07-30

**Authors:** Yubo Wang, Xinyu Li, Feng Gao

**Affiliations:** Clinical Laboratory of China-Japan Union Hospital, Jilin University, Changchun, China

**Keywords:** chronic obstructive pulmonary disease, microbiota, gut-lung axis, prevention and treatment, fecal microbiota transplantation, nutritional therapy

## Abstract

The pathogenesis of chronic obstructive pulmonary disease (COPD) is complex and affects multiple systems. This article focuses on COPD and elaborates on the roles of the lung and gut microbiota as well as preventive and therapeutic strategies. Innovatively, this article reveals the impact of the connection between the lung and gut microbiota via the gut-lung axis on COPD, clarifies the association between changes in the lung microbiota and clinical features, enriches the understanding of the correlation between gut dysbiosis and COPD, breaks through the limitations of single-organ research, and opens up a completely new path for uncovering the underlying pathogenesis of COPD. In terms of prevention and treatment, gut microbiota-targeted therapies (fecal microbiota transplantation, probiotics and prebiotics) provide new ideas and evidence. Research on dietary factors (vitamins, dietary fiber) helps with precise nutritional interventions and highlights the significance of dietary adjustments. The multi-target actions of natural compounds integrate traditional and modern medicine and lay the foundation for the development of new approaches, which is of great significance and value for COPD research, clinical translation, and the improvement of patient health.

## 1 Introduction

Chronic obstructive pulmonary disease (COPD) is a common preventable and treatable disease characterized by persistent respiratory symptoms and airflow limitation caused by airway and/or alveolar abnormalities resulting from toxic particles or gases (1), and is characterized by clinical features including chronic cough, sputum, or dyspnea, asthma, and chest tightness (2). COPD affects nearly 400 million people worldwide, and its incidence continues to rise each year, resulting in an enormous socioeconomic burden (3). COPD is a complex disease with multiple sub-phenotypes affecting not only the lungs, but also the cardiovascular, gastrointestinal, and immune systems (4–6), and the underlying mechanisms are incompletely understood, with possible causes ranging from exposure to cigarette smoke to genetic predisposition to social and environmental factors (7).

COPD is known to be associated with reduced diversity of the normal lung microbiota and replacement of resident flora by potentially pathogenic microorganisms (8). In healthy lungs, the predominant phyla are *Bacteroides and Firmicutes* (9). In turn, patients with COPD have an increased number of *Proteobacteria* (10, 11). Studies have shown that dysbiosis of the gut flora is associated with a variety of local and distant chronic diseases (12). A balanced microbial community in the gut is important for immune function and health (13). For example, COPD patients tend to have an altered gut microbiome, which appears to be associated with reduced microbial diversity, an increase in *Firmicutes, Prevotella, and Streptococcus*, and a decrease in Bacteroidetes (14).

Gut and lung microbes interact with each other through the blood or lymphatic system. Immune cells and metabolites produced by gut bacteria can move through the circulatory system to stimulate immune responses in the lungs, while the lung microbiota can induce microbial changes in the blood and gut (15, 16). The crosstalk between the gut microbiome and the lungs is known as the gut-lung axis (17). For example, patients with inflammatory bowel disease (IBD) exhibit changes in the composition of the gut microbiota and decreased lung function, while patients infected with influenza viruses often exhibit gastrointestinal symptoms (2, 18). The gut-lung axis plays an important role in shaping local cellular function, directing host immune responses inside and outside the lungs, and in the development of COPD (17, 19). However, the underlying mechanisms remain unclear. Therefore, we explored the role of lung and gut microbiota in COPD, the mechanisms and recent advances in treatment.

## 2 Microbiota and COPD

### 2.1 Lung microbiota and COPD

Traditionally, lungs are considered sterile spaces, but in fact, they have their own microbial community composed of bacteria, viruses, and fungi (20). A large amount of published evidence suggests that high-throughput sequencing can be used to detect the microbial diversity in the lungs of healthy individuals. The lung microbiota can be classified through metagenomic next-generation sequencing and 16S rRNA gene sequencing of DNA extracted from samples collected via sputum, lung tissue, or bronchoalveolar lavage fluid (BALF) (21, 22). Summarizing published studies, at the phylum level, *Proteobacteria, Firmicutes, and Bacteroidetes* are the most common genera in COPD patients, while at the genus level, *Pseudomonas, Streptococcus, Prevotella, Fusobacteria, and Veillonella* are the main genera. However, the promoting effect of potential pathogens including *Haemophilus and Neisseria* is relatively small. The microbial community in the upper respiratory tract of the lungs is similar to that in the oral cavity, while the more unique microbial composition is found in the lower respiratory tract (23).

Maintaining the health of the body is also a function of the microbiota, and changes in the composition of the microbiota due to genetic, lifestyle, and environmental factors can lead to the onset of respiratory dysfunction, such as COPD (24). The current research on the relationship between lung microbiota and COPD has attracted people’s attention. The characteristics of COPD are persistent inflammation of the lower respiratory tract, dysfunction of mucociliary activity, and pulmonary emphysema, ultimately leading to irreversible airflow limitation (25). Patients often experience difficulty breathing, coughing, sputum, and changes in respiratory composition due to long-term exposure to cigarette smoke (CS) or pollutants (26). CS mainly affects microbial composition through hypoxia and increased formation of bacterial biofilms (27). A good composition of pulmonary and respiratory microbiota can help the host establish a protective environment, create a hostile environment for pathogen customization, and maintain host homeostasis (28). Microorganisms enter the lungs through the respiratory tract and colonize, reducing factors that regulate their elimination. The immune imbalance caused by these different fixed values may lead to pulmonary dysfunction, resulting in the pathogenesis and clinical course of the disease (28). Thus, changes in the local microbial composition of the respiratory system are associated with the progression of airway inflammation and COPD.

The frequency of COPD is directly related to respiratory infections and the proliferation of pathogens. During the inflammatory process of COPD, alveolar macrophages and neutrophils produce reactive oxygen species (ROS) and nitrogen substances (29). These increase in the levels will lead to oxygen consumption of lung mucus, transforming the lung microenvironment from aerobic to anaerobic, resulting in an increase in anaerobic bacteria. *Pseudomonas aeruginosa* is one of the anaerobic bacteria present in the lungs and belongs to the genus *Gamma Proteobacteria* (30). They can live under aerobic and anaerobic conditions and become pathogenic in certain situations. In fact, once *Pseudomonas aeruginosa* infection is confirmed, it is associated with high mortality rates as it can lead to acute lung injury and respiratory distress syndrome, accompanied by treatment complications (31). Another study also showed that patients with frequent deterioration had an increase in *Pseudomonas*, *Moraxella*, and *anaerobic cocci* compared to those without deterioration. The most widespread viruses found in COPD are human rhinovirus and influenza virus (32–34). But there are also studies claiming that cytomegalovirus and Epstein-Barr virus have been detected in the lungs of COPD patients (35, 36). In addition, fungal pathogens are relatively increased in patients with chronic obstructive pulmonary disease, including *Candida*, *Aspergillus*, *Penicillium*, *Cladosporium*, etc. Although the composition of the pulmonary microbiome in COPD is well understood, a description of the pulmonary microbiome alone is insufficient to gain a deeper understanding of its mechanisms (33, 37). A 2020 study suggests that the regulation of microbiota on the severity of chronic obstructive pulmonary disease may be related to NLRP3 inflammasomes. NLRP3 inflammasomes in the lungs are activated by pathogens to promote the recruitment of inflammatory cells, regulate the immune response of the gastrointestinal and respiratory tracts, and maintain the symbiotic bacteria and their metabolites on the upper skin barrier, leading to immune cell activation and lung inflammation to protect the human body (38).

### 2.2 Gut microbiota and COPD

The human gut microbiota is involved in a variety of interactions that affect host health throughout the host’s life cycle (39). Gut microbiota regulate many metabolic processes in the host, including energy homeostasis, glucose metabolism and lipid metabolism (40). The gut microbiota evolved with the host and is an integral part of the body (41). From infants to the elderly, from primitive tribes to modern societies, there is growing evidence that key physiological roles of the gut microbiota are implicated in the pathogenesis of a variety of human metabolic, immune, and neurological disorders (42). Several studies have explored the relationship between gut flora and lung disease. For example, a previous study explored recent advances in gut flora and its metabolites in typical lung diseases (**Table 1**), such as pulmonary hypertension, chronic obstructive pulmonary disease and lung cancer (43). In addition, studies have shown that the gut microbiome of patients with COPD exhibits unique overall microbial diversity and composition, as well as lower levels of short-chain fatty acid (SCFA), especially in patients with lower lung function. The changes in the gut microbiota of COPD patients are associated with airway inflammation in mice and the accelerated progression of COPD (44).

**Table 1:** Relationship between gut microbiota and COPD.

Author and year	Variables	Summary findings	Research contents	Outcomes
Shen, Huan-Ting et al. (2024) (52)	Fecal samples from COPD mice	*Bifidobacterium* and *A. muciniphila* levels decreased	oral administration of the probiotic composition containing *L. reuteri* GMNL-89 and *L. paracasei* GMNL-133	Improving emphysema and inflammation
Laiman, Vincent et al. (2024) (53)	Microbiome Data in COPD mice and patients	*Actinobacteria* decreased and *Firmicutes* increased	Disorder of the microbiome in the lungs and the intestines	Increase in markers of lung inflammation
Bowerman, Kate L et al. (2020) (54)	Fecal samples from COPD patients and healthy controls	*Streptococcus*, *Rothia*, *Romboutsia*, *Intestinibacter*, *Streptococcaceae* and *Escherichia* increased; *Bacteroides, Roseburia, Lachnospira, and unnamed Ruminococcaceae* decreased.	The microbiome and metabolome revealed that there were 146 differences in bacteria between the two groups.	Reduced lung function
Lai, Hsin-Chih et al. (2022) (45)	Colon, lung tissues and fecal samples from COPD mice	*Lachnospirillaceae* increased *Bacteroidales* and *Ruminococcaceae* decreased	Single-cell RNA sequencing and serum metabolomics analysis were conducted to identify host response molecules. Fecal microbiota transplantation can restore the occurrence of COPD.	Improve inflammation and lung function
Chiu, Yu-Chi et al. (2022) (55)	Stool samples from COPD patients	*Alloprevotella* increased	Microbiome sequencing of COPD patients after one-year follow-up	Reduced lung function
Sun, Zhe et al. (2020) (47)	Feces and sputum samples from AECOPD patients	*Firmicutes* and *Bacteroidetes* had high levels *Acidobacteria* and *Cyanobacteria* had low levels	The dynamic changes of the bacterial microbiome in the gut and lungs	Bacterial colonization leads to decreased lung function and increased inflammation.
Yan, Jiali et al. (2024) (56)	Fecal samples from stable COPD samples, AECOPD samples, and healthy individuals.	The elevated levels of Lachnoclostridium and Prevotella may suggest an acute exacerbation of COPD.	16S sequencing analysis of intestinal microorganisms	There are significant differences in the bacterial community composition among AECOPD patients, COPD patients and healthy controls.

In recent years, more and more studies have found that intestinal microbial disorders in COPD patients (**Table 1**). Lai et al. reported that the composition of the gut microbiota has an important impact on the pathogenesis of COPD and that fecal microbiota transplantation can restore the pathogenesis of COPD. In addition, A commensal symbiotic bacterium, *Parabacteroides goldsteinii*, was isolated and shown to improve chronic obstructive pulmonary disease (45). Wu et al. reported a decrease in the relative abundance of *Firmicutes and Actinomycetes* and an increase in the relative abundance of *Bacteroidetes and Proteobacteria* in AECOPD compared to COPD and NS. Within the overall structure of the intestinal flora, *Bacteroides and Bifidobacterium* were specific distinguishing bacteria with high relative abundance in the intestinal flora of AECOPD and stable COPD patients, respectively (46). Studies have reported increased levels of *Firmicutes* and *Bacteroidetes* and lower levels of *Acidobacteria* and *Cyanobacteria* in intestinal samples from patients with COPD (47). Wang et al. carried out a study involving 40 COPD patients and 40 healthy controls and reported that the gut microbiota was highly correlated with the immune status of COPD patients (48).

There are differences in the characteristics of intestinal flora in patients with COPD, suggesting that intestinal flora may affect the treatment and prognosis of COPD. Since the gut microbiota plays a role in regulating immune responses and systemic inflammation, the gut microbiota also plays an important role in the treatment of COPD. COPD is a systemic inflammatory disease. Intestinal microorganisms release various metabolite products and interact with human tissues, which plays an important role in reducing systemic inflammation (49). In recent years, a large number of studies have shown that there is a bidirectional regulatory relationship between gut microbiota and lung inflammation. SCFA is the most deeply studied bacterial metabolite in the gut and SCFA can modulate metabolic programming in LPS-exposed alveolar macrophages, which helps to maintain lung immune metabolism, highlighting the potential clinical application of SCFA in the treatment of diseases such as COPD in the study (50). Li et al. reported that BSP alleviated COPD by regulating intestinal flora in mice, and BSP alleviated COPD by improving pulmonary ventilation function, reducing proinflammatory cytokine levels, and reducing oxidative stress (51). It has been reported that *Firmicutes* and *Actinobacteria* have a highly significant association with blood inflammatory indicators in COPD, which suggests that these bacterial taxa may participate in the occurrence and development of COPD by aggravating systemic inflammation (46). The imbalance of microbial flora plays an important role in the occurrence and development of COPD, affecting the intestine and promoting the injury of immune response (36).

However, the role of gut microbiome in the occurrence and development of COPD needs to be further explored. In addition, the role of the gut microbiome in modulating the effectiveness of COPD treatment should be further analyzed.

## 3 Gut–lung axis and COPD

**Figure 1:**
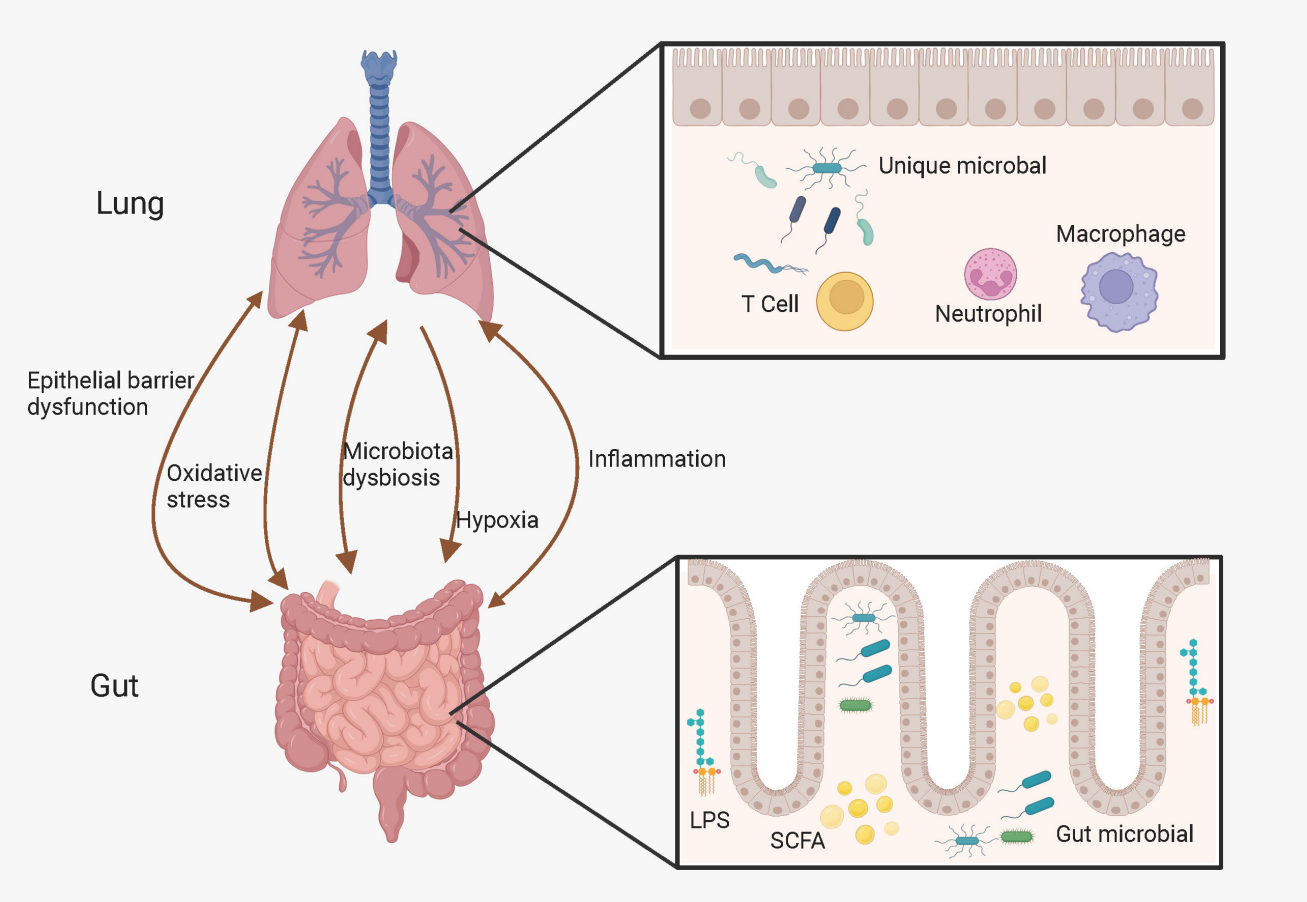
Possible link between gut-lung axis and COPD. The gut-lung axis is believed to operate bidirectionally and this intricate interplay is facilitated by a complex network involving hypoxia, inflammation, oxidative stress, microbiota dysbiosis, and epithelial barrier dysfunction. (Created with Biorender.com).

Although the gastrointestinal and respiratory tracts have different distributions and functions, they share the same embryonic origin and common entrance, and both dominate the body’s interaction with the external world. Thus, these two parts may interact with each other in several ways. Studies have shown that there is a bidirectional crosstalk between the gut and the lungs, called the “gut-lung axis” (57–59). In addition, communication between the gut and lungs may occur through systemic inflammation, oxidative stress, epithelial barrier dysfunction, hypoxia, and dysbiosis of the gut microbiota and alterations in its metabolites (57, 60), as **Figure 1**.

Cigarette smoke is a major cause of COPD. Cigarette smoke-induced oxidative stress and pro-inflammatory responses disrupt airway epithelial barrier function and lead to impaired gas exchange in the lungs, which promotes systemic hypoxia and induces intestinal hypoxia, inflammation, mucosal angiogenesis and epithelial cell death, as well as reduced intestinal barrier function, which leads to an increased risk of gastrointestinal disease (61, 62). In addition, studies have shown that patients with COPD have increased intestinal permeability at rest and that performing activities of daily living (ADLs) leads to intestinal epithelial cell damage (6). The systemic inflammation of COPD will also have an impact that affects small intestinal absorptive function activity (63). In chronic obstructive pulmonary disease, hypoxia-inducible factor (HIF)-1α expression is increased in the small intestine due to increased oxidative stress and apoptosis, ultimately leading to disruption of the small intestinal tight junction (TJ) (64). And the antioxidant ebselenalin may improve cigarette smoke-induced gastrointestinal dysfunction (65).

Dysbiosis of the intestinal flora and alterations in its metabolites are closely associated with the progression of COPD, while COPD also affects the intestinal flora and its metabolites (66). The gut microbiota plays an important role in the pulmonary immune response by producing metabolites such as SCFA and initiating immune cells that act on the lung mucosa via the lymphatic and circulatory systems. When the gut flora is dysbiotic, it not only undermines intestinal immunity, but may also promote the development of COPD (67). A recent human study also found that the bacterial species in the fecal microbiome of COPD patients and healthy controls were different. Not only the respiratory tract, but also the structure of the intestinal microbiome may be involved in controlling the development of COPD. Therefore, fecal microbiota transplantation (FMT) can be used to restore the treatment of chronic obstructive pulmonary disease (45). Studies have shown that COPD patients have dysbiosis of the gut flora and reduced levels of SCFA, which ultimately leads to increased airway inflammation and COPD progression (44). In addition, when rats were exposed to cigarette smoke, the levels of organic acids such as acetic acid, propionic acid, butyric acid and valeric acid in the cecum were significantly reduced, the population of *bifidobacteria* was significantly reduced, and the intestinal environment of the rats was altered, which led to intestinal diseases (68). *Pentosaccharomyces cerevisiae SMM914* was found to be a probiotic that produces beneficial metabolites and modulates the gut microbiota, which in turn attenuates oxidative stress-induced lung inflammation and lung damage in COPD via the gut-lung axis (69). In addition, Shen et al. found that oral administration of a probiotic combination containing *Lactobacillus Royale GMNL-89* and *Lactobacillus paracasei GMNL-133* improved emphysema and lung inflammation in LPS/elastase-induced COPD mice. The mechanism may be that the probiotic intervention alters the diversity of microbial communities in the gut, which in turn alters the short-chain fatty acid (SCFA) content in the gut, further regulating the lung microbiota and ameliorating lung dysfunction and inflammation via the gut-lung axis (52).

## 4 Prevention and treatment of COPD

**Figure 2:**
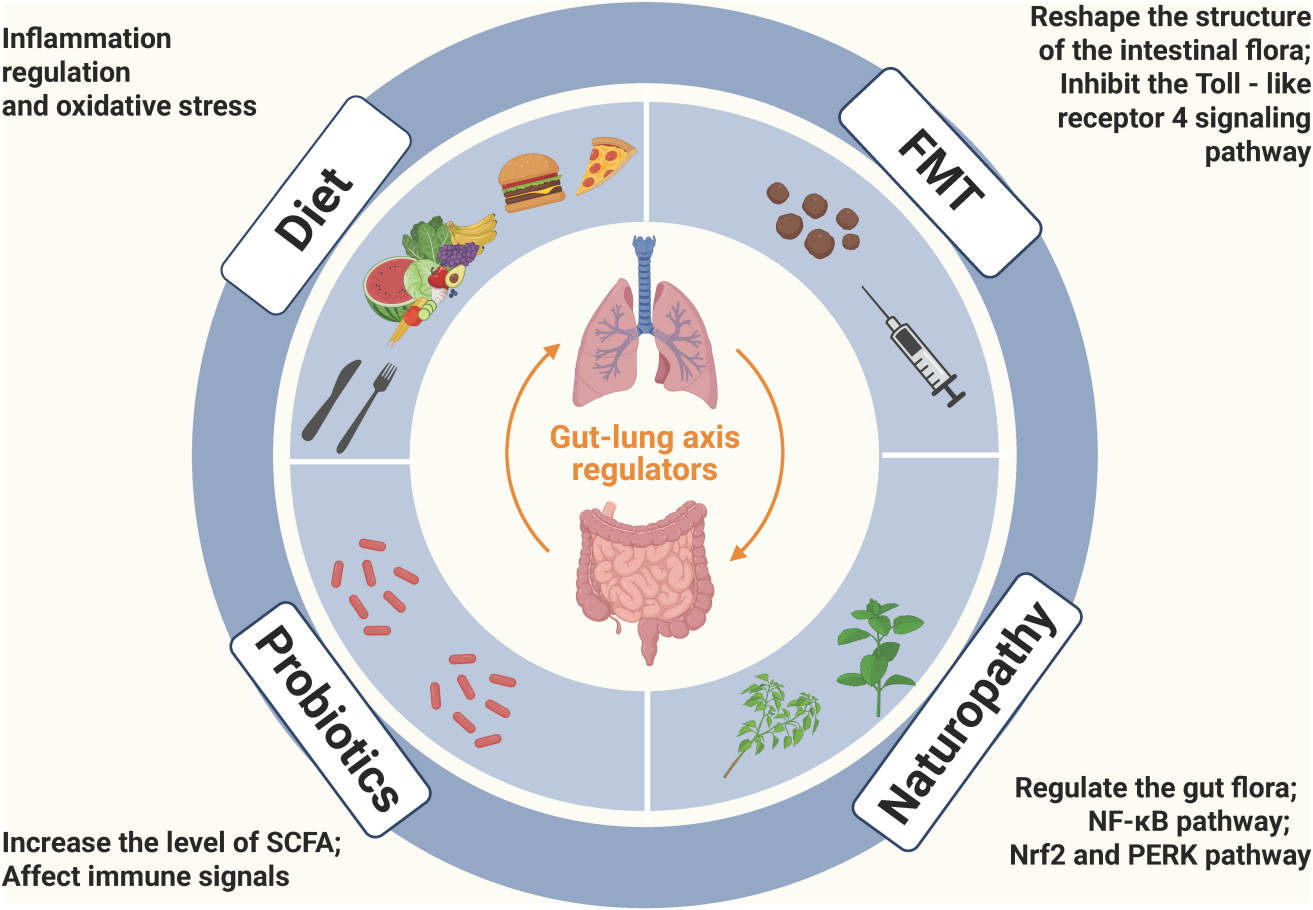
Potential treatment options for COPD based on gut-lung axis: FMT, Probiotics, Diet, and Naturopathy (Created with Biorender.com). FMT can reshape the structure of the gut microbiota, improve airway inflammation and emphysema in COPD mice, and in particular, the lipopolysaccharide of *P. goldsteinii* exerts an anti-inflammatory effect by acting as an antagonist of the toll-like receptor 4 signaling pathway; Probiotic strains can relieve respiratory symptoms, reduce lung inflammation and improve lung function through various mechanisms such as regulating the balance of cytokines, increasing the level of short-chain fatty acids, and affecting the immune signaling pathway; Dietary factors play a significant role in COPD, especially the intake of antioxidant nutrients, and they mainly affect the progression of COPD through the mechanisms of inflammation regulation (such as ω-3 fatty acids and flavonoids regulating the inflammatory response) and oxidative stress (such as vitamin C and vitamin E scavenging free radicals); Active compounds derived from Chinese herbal medicines and other natural sources can regulate the gut microbiota, enhance the gut barrier, inhibit inflammation-related signaling pathways, and improve oxidative stress to alleviate the symptoms of COPD.

In terms of the prevention and treatment of COPD, a variety of strategies have been explored, yet each has its own challenges. This chapter summarizes the current prevention and treatment strategies for COPD, including targeted therapies based on the gut microbiota (fecal microbiota transplantation and probiotics or prebiotics), dietary habits (dietary fiber and vitamins), and natural therapies (active compounds from Chinese herbal medicines and natural sources), as shown in **Figure 2**.

### 4.1 Fecal microbiota transplantation

Sequencing technologies of the gut microbial ecosystem have assisted researchers in exploring the crucial roles that the gut microbiota plays in human health and diseases (70). The manipulation of the gut microbiome holds great potential for reducing the occurrence or severity of various human conditions and diseases (71). Fecal microbiota transplantation (FMT), as an intervention for the gut microbiome, has rapidly drawn the attention of the scientific and clinical fields (72). FMT refers to the transplantation of minimally processed feces from a healthy donor into the recipient’s gut for the treatment of diseases associated with changes in the gut microbiota. FMT was initially used for the treatment of gastrointestinal-related diseases. Clinical trials of FMT have included diseases such as Clostridium difficile infection (73), ulcerative colitis (74), and diarrhea-predominant irritable bowel syndrome (75). Subsequently, clinical trials of FMT have also involved non-infectious diseases such as cancer (76), adjuvant immunotherapy (77), metabolic syndrome (78), Parkinson’s disease (79), non-alcoholic fatty liver disease (80), and type 2 diabetes (81). This indicates that FMT, as a means of treating diseases starting from the gut microbiota, has been favored by clinical researchers and they are committed to developing innovative treatment models for diseases. In situations where traditional treatment methods may face limitations, FMT has opened up a completely new clinical treatment approach.

The composition of the gut microbiota can significantly affect the development of CS-induced COPD, and fecal microbiota transplantation from mice can improve COPD (45). The mechanism of this effect is that the lipopolysaccharide of *P. goldsteinii* exerts an anti-inflammatory effect by acting as an antagonist of the toll-like receptor 4 signaling pathway. After receptor mice were transplanted with feces from COPD patients for 28 days, they exhibited elevated lung inflammation. And when the mice were simultaneously exposed to fecal transplantation from COPD patients and biomass fuel smoke for a total of 20 weeks, accelerated decline in lung function, severe emphysematous changes, airway remodeling, and excessive mucus secretion were observed (44). In addition, FMT or a high-fiber diet (HFD) alleviates the development of cigarette smoke-induced emphysema through local and systemic suppression of inflammation and changes in the composition of the gut microbiota (82). Therefore, we can artificially intervene in the gut microbiota to regulate the progression of COPD. To date, there have been no clinical trials of fecal microbiota transplantation in COPD patients to determine the effectiveness of this method.

Although fecal microbiota transplantation (FMT) has a recognized role in the treatment of recurrent Clostridium difficile infection (CDI), its wide dissemination is restricted by several obstacles, including the lack of specialized centers, difficulties in donor recruitment, and the complexity associated with regulatory and safety monitoring, which makes it difficult to define its precise function (83). Therefore, evaluating its clinical efficacy and safety in different disease contexts has become a rather challenging task, and there are still certain limitations in its extensive clinical application. The primary concern in conducting FMT clinical trials is to ensure the safety of the FMT product, and the second is to select the “optimal” donor. However, it is difficult to define an ideal donor according to the indications and the composition of the gut microbiota. There are significant differences in donor exclusion criteria and screening levels. Affected by different global regulations and the uncertainty of FMT safety, most studies have not reported donor selection or testing in detail (84). In allogeneic hematopoietic stem cell transplantation, autologous frozen fecal transplantation can reduce the risk of infection and enhance the curative effect due to better engraftment when patients’ intestinal microbiota is damaged after antibiotic treatment (85). Currently, in clinical practice and research, the selection of donors mainly relies on three approaches: traditional health screening, assessment based on microbiota diversity, and personalized matching (86). The traditional health screening protocol, which focuses on excluding the risks of infectious diseases and underlying medical conditions, is easy to operate and highly safe, yet its failure to consider the functions and diversity of the microbiota may result in poor post-transplantation microbiota colonization (87). The screening protocol based on microbiota structural diversity, which selects donors by detecting microbial richness and beneficial bacteria abundance, shows potential in enhancing transplantation efficacy for complex disease treatment but suffers from the lack of a unified standard (88, 89). Although the personalized matching protocol can accurately screen donors according to the disease needs of the recipients, it faces problems such as high screening difficulty and long screening time. Through comparison, it can be seen that each protocol has its own advantages and disadvantages in different application scenarios, which further highlights the complexity of defining an “optimal donor” that requires the integration of multi-dimensional criteria (90). Given that the complexity of the microbial community in the natural course of diseases leads to the fact that a single taxon cannot stably predict the clinical outcome, future research is required to explore new matching models by means of methods such as hierarchical analysis or machine learning, so as to accurately screen out donors that are suitable for specific recipients, thereby improving the clinical effectiveness of FMT (91, 92). Despite the limitations of FMT, it remains one of the most important tools for investigating the causal contribution of the microbiome to a series of chronic diseases at present (93). By using sophisticated bioinformatics techniques and machine learning algorithms to link detailed phenotypes of gut microbiome, plasma and/or fecal metabolites, and patient phenotypes, promising leads can be identified, for example, Zhao et al. identified the core microbiome characteristics of healthy organisms, providing a powerful framework for the development of microbiome-based targeted therapies (94).

### 4.2 Probiotics and prebiotics

Probiotics refer to bacteria that can promote the health of the organism. The complex relationship between them and human health extends beyond their traditional function in gut health and has attracted considerable interest due to their extensive potential in disease treatment (95). Probiotic strains promote the maintenance of a favorable gut environment by promoting a healthy gut microbiota and are frequently used in the treatment of gastrointestinal tract (GIT)-related issues (96). The probiotic strains that can be used in infant food currently include strains such as *Bifidobacterium* and *Lactobacillus*. In addition, probiotics can effectively maintain gut epithelial regeneration and homeostasis and repair gut damage after pathological injury (97), enhance gut barrier function and prevent the leakage of pro-inflammatory substances (98). Besides GIT-related problems, the therapeutic effect of probiotics on COPD has also been studied (99). An early clinical study indicated that short-term intake of multi-species probiotics had little impact on the gut microbiota of COPD patients (100), suggesting that short-term probiotic intake has no significant effect and may require longer-term or more precise interventions. Another clinical study demonstrated that *Lactiplantibacillus plantarum KC3* and *Leonurus japonicus* could relieve respiratory symptoms and improve lung function both *in vitro* and *in vivo* (101). The results of a cross-sectional study based on the NHANES database showed that, after adjusting for factors such as gender, age, education level, activity intensity, and other diseases, the consumption of probiotics, prebiotics, or yogurt was associated with a lower incidence of COPD (102), indicating that the consumption of probiotics, prebiotics, or yogurt may play a beneficial role in the prevention of COPD.

The great potential of probiotics in improving lung conditions and treating COPD has also been verified in mouse experiments (103). Oral administration of *Lactobacillus rhamnosus* can alleviate cigarette smoke-induced COPD, and the underlying mechanism may be closely related to restoring the balance between pro-inflammatory and anti-inflammatory cytokines in bronchial epithelial cells (104). A synbiotic mixture (containing *Bifidobacterium breve M16-V*, oligosaccharides, and pectin) has a positive effect. It can enhance the production of short-chain fatty acids and contribute to the improvement of lung health (105). Similarly, a combination of lactic acid bacteria (LAB) consisting of *Lactobacillus reuteri GMNL-89* and *Lactobacillus paracasei GMNL-133* also has the ability to increase the level of short-chain fatty acids. Through this pathway, it can reduce lung inflammation and thus improve the emphysema condition in mice with COPD (52). *Lactobacillus rhamnosus* plays an important role in the prevention and treatment of lung diseases. It can induce antiviral signaling related to IFN-γ-secreting viral sensors (106). In addition, in macrophages exposed to cigarette smoke, it can attenuate the cytokine storm related to NF-κB, which opens up a new role for it in preventing the exacerbation of COPD (107). Moreover, in a mouse model of asthma-COPD overlap syndrome, *Lactobacillus rhamnosus* exhibits multifaceted improvement effects. It can reduce the proportion of white blood cell populations, relieve bronchial hyperresponsiveness, decrease the level of pro-inflammatory cytokines, and thus improve airway remodeling. At the same time, it can increase the proportion of gut probiotics and ultimately effectively improve the overall condition of asthma-COPD overlap syndrome (108), and the mechanism may depend on the GPR43 receptor of macrophages (109). Oral administration of the probiotic *Pediococcus pentosaceus SMM914 (SMM914)* can enhance the abundance of gut probiotics and strengthen the tryptophan-melatonin pathway, increasing endogenous anti-inflammatory factors such as 6-hydroxymelatonin and secondary taurine in the lung environment, reducing the polarization of macrophages to the M1 phenotype, and finally alleviating oxidative stress in COPD mice (69). There is also research analyzing that the administration of the probiotic *Bifidobacterium longum subsp. longum* can alleviate cigarette smoke-induced lung inflammation regardless of its acetate-producing ability (110), suggesting that *Bifidobacterium* can directly interact with the host’s immunity, independent of SCFAs.

### 4.3 Diet

The role of dietary factors in the prevention of COPD has been recognized, and the evidence regarding antioxidant nutrients, vitamin and fiber intakes is particularly crucial. Using nutritional means to promote lung health has opened up a new path for intervening in COPD. Diet affects COPD mainly through the following mechanisms (111): First, the inflammation regulation mechanism. Certain food components, such as deep-sea fish rich in ω-3 fatty acids and fruits and vegetables containing flavonoids, can regulate the body’s inflammatory response, reduce the production of inflammatory mediators, thereby alleviating lung inflammation and tissue damage in COPD patients and delaying the progression of the disease (112, 113). Second, the oxidative stress mechanism. Antioxidant nutrients such as vitamin C, vitamin E, and β-carotene can scavenge free radicals and prevent lipid peroxidation reactions respectively (114, 115), neutralize free radicals caused by smoking, air pollution, etc., reduce the damage of oxidative stress to the lungs, and play a preventive and adjuvant therapeutic role. A national health and nutrition examination in South Korea indicated that the intakes of carbohydrates, proteins, fiber, thiamine, riboflavin, niacin, and vitamin C were significantly associated with a reduction in disease severity in elderly men (≥ 60 years old) (116). A single-blind randomized trial involving 46 COPD patients showed that taking a specific whey drink reduced IL-6 concentration and improved fat-free mass index, body protein, grip strength, and health-related quality of life (117). Therefore, clinicians can consider incorporating nutritional intervention into the comprehensive treatment plan when treating COPD patients and pay attention to the impact of patients’ nutritional intake and body composition changes on the disease process.

#### 4.3.1 Dietary fiber

The evidence that consuming whole fruits at an adequate level is beneficial to health has been steadily growing, especially regarding their prebiotic effect of bioactive fiber (118). Promoting the consumption of vegetables and fruits through nutrition and health policies is a desirable strategy for alleviating the burden of several chronic diseases in Western societies (119). Some studies have found that the intake of dietary fiber is related to the prevalence of inflammatory airway diseases, and a higher intake of dietary fiber is associated with a reduced mortality rate among participants with chronic inflammatory airway diseases (120). Moreover, in chronic obstructive pulmonary disease, the therapeutic potential of dietary fiber stems from the gut - lung axis (121). This is because the short-chain fatty acids produced by the fermentation of dietary fiber connect the microbial communities in the lungs and intestinal mucosa through the gut - lung axis and exert anti-inflammatory and immunosuppressive effects in the lungs (122).

There is a negative correlation between dietary fiber intake and COPD, and there is suggestive evidence of a positive correlation with lung function (123, 124), especially in male smokers or middle-aged male populations (125). This may be related to white blood cells (126). However, a high dietary fiber intake may not change the association between COPD and cognitive impairment (127). Nevertheless, in a follow-up experiment on women, subjects with a long-term high dietary fiber intake (≥ 26.5 g/d) were associated with a 30% lower risk of COPD (128). The associations between dietary fibers from different sources and COPD may vary. An earlier study indicated that cereal fiber may be more important in preventing COPD risk, independent of fiber from fruits and vegetables (129). It has been found that for every 10 grams increase in daily fruit fiber intake, there is a 37% difference in the risk of developing COPD when compared to those with lower intakes, while the same increase in total dietary fiber and cereal fiber intakes, the differences in risk are 26% and 21% respectively (130), and the consumption of processed meat is associated with a higher risk of COPD (131). The reason for the different conclusions may be that with the progress of society and the development of the economy, the source of dietary fiber may shift from cereal fiber to fruit fiber. There is also research showing that, whether among dairy farmers or smokers without occupational exposure, there is no significant difference in the dietary patterns of COPD patients (132), that is, COPD patients do not change their dietary patterns due to the disease.

It has been discovered in epidemiology and further verified in animal experiments that a high-fiber diet exerts beneficial effects by modulating the gut microbiota and metabolites and has the potential to treat emphysema (133). Short-chain fatty acids are the fundamental mediators of the gut-lung axis (134). There is a close association between the attenuation of fecal SCFA levels and the increase in neutrophilic lung inflammation in COPD (105). The microbial butyrate derived from dietary fiber may enhance the acetylation of the NFIL3 promoter through histone deacetylase (HDAC) inhibition, thereby increasing its expression and ultimately suppressing cytokine production in ILC2, which plays a role in improving COPD (135). Given the increasing burden of COPD, more attention should be paid to diet quality as an alterable factor in the occurrence and progression of the disease in COPD patients.

#### 4.3.2 Vitamins

Serum antioxidant vitamin concentrations decrease during acute exacerbations of COPD and are accompanied by an increase in oxidative stress (136–139). Studies have shown that serum vitamin A concentration may be important in preventing the progressive decline in lung function parameters that lead to COPD (137). In addition, an early clinical follow-up experiment demonstrated that vitamin A supplementation can improve lung function outcomes in mild COPD and further supports the relationship between vitamin A and lung health (140), suggesting that sufficient intake of vitamin A is crucial in early lung development, alveolar formation, tissue maintenance, and regeneration (141). Vitamin C has the effect of relaxing tracheal smooth muscle (142) and can affect the development of COPD by modulating the PI3K/AKT signaling pathway (143). A high dietary intake of vitamin C or vitamin C-rich foods may slow the rate of loss of lung function in adults, thereby contributing to the prevention of chronic obstructive pulmonary disease (144, 145), and this protective effect is independent of smoking history (146). A higher intake of vitamin E reduces the risk of all-cause mortality and mortality from chronic lower respiratory tract diseases (CLRD) in COPD patients (147), and this association is independent and not related to gender (145, 148). The related mechanism of action may be closely related to EGFR/MAPK and the inhibition of the translocation of phosphorylated STAT3 into the nucleus (149). The vitamin E subtype γ-tocotrienol has anti-inflammatory and antioxidant effects and can be a potential drug for the treatment of COPD (150). However, other studies have shown that supplementation with 400 IU of vitamin E on top of the standard treatment for COPD only increased certain endogenous antioxidant levels in patients but failed to provide sufficient additional clinical benefits (137, 151). Especially after calibrating the effect of vitamin C, vitamin E had no additional independent effect on FEV1 or FVC (152). The reason for this contradictory conclusion is the different doses of vitamin E ingested. When the intake of vitamin E is 600 IU, the risk of chronic lung disease in women is reduced by 10% (148).

In asthma and chronic obstructive pulmonary disease, the metabolism of vitamin D is dysregulated. Compared with women, men have significantly lower vitamin D levels (139, 153). Related studies have shown that a considerable proportion of patients with COPD have insufficient vitamin D levels (153), and vitamin D deficiency is associated with a decline in lung function and an increased risk of acute exacerbations of COPD (138, 154), which may be related to a decrease in glutathione peroxidase 4 (GPX4) and an increase in iron parameters (155). Previous studies have indicated (156, 157) that the 25(OH)D level may be a useful marker for adverse outcomes in COPD and is correlated with the improvement of lung function markers. However, it has no association with the prevalence of respiratory diseases (158). It should be emphasized that the causal relationship between COPD and vitamin D deficiency remains unresolved (159, 160). However, from a genetic perspective, evidence shows that for every 1 standard deviation increase in 25(OH)D level, the relative risk of COPD is reduced by 57.2% (161), and it has a 22% mediating effect on diet-induced inflammation and all-cause mortality in COPD patients, showing a nonlinear L-shaped correlation (162). Vitamin D supplements have the effect of reducing in-hospital mortality in severe COPD patients, and the effect is more significant in the female patient population (163). The mechanism of action may be through the regulation of FeNO and 25(OH)D levels, downregulating inflammatory factors, thereby reducing the degree of inflammation (164). However, studies on high-risk individuals have found that antioxidant vitamins did not significantly reduce the mortality and incidence of any type of vascular disease, cancer, or other major outcomes within 5 years (165). In addition, the results of a multicenter, double-blind, randomized controlled trial showed that for COPD patients with vitamin D deficiency, vitamin D supplements did not reduce the exacerbation rate of their condition (166), and the supplement did not improve lung function in all individuals, but it was beneficial for regular smokers (167, 168). Animal and cell experiments have demonstrated that vitamin D has a positive effect on preventing the exacerbation of COPD infections, which helps to enhance the defense ability of human bronchial epithelial cells against air pollution exposure (169), and can reduce the exacerbation of infections by regulating the expression of antibacterial peptides (170) and inhibiting related cellular inflammation and fibrosis responses (171). Vitamin D exerts its role in lung-related diseases mainly through the vitamin D receptor axis. Specifically, the vitamin D3-vitamin D receptor axis can inhibit emphysema by maintaining the homeostasis and function of alveolar macrophages (172). In transgenic mouse experiments, it was found that the lung tissue of mice overexpressing the vitamin D receptor (VDR) exhibits anti-inflammatory properties, which fully demonstrates the importance of the vitamin D receptor in regulating lung inflammation (173). In addition, during the process of pulmonary fibrosis, the upregulation of the fibroblast vitamin D receptor can be regarded as a self-protection mechanism of the body, because it can effectively limit the proliferation and activation of fibroblasts by inhibiting the JAK1/STAT3/ER stress pathway, thereby affecting the development process of pulmonary fibrosis (174).

### 4.4 Natural therapies

Active compounds from Chinese herbal medicines and other natural sources possess anti-inflammatory, antioxidant, anti-tumor, and immunomodulatory effects, endowing them with significant value in the prevention and treatment of respiratory diseases (175). A great number of active compounds derived from Chinese herbal medicines and other natural sources play a non-negligible role in the prevention and treatment of COPD. Active compounds from natural sources play an essential role that cannot be overlooked in the regulation of the gut and related functions in COPD. For instance, *Seabuckthorn Wuwei Pulvis* (SWP) can effectively modulate the gut microbiota, during which the production of SCFAs is increased, and the gut barrier function is strengthened simultaneously (176). Moreover, Magnolol can enhance the gut barrier function in COPD and improve the gut microecological environment, which further exerts a positive impact on lung function, leading to the improvement of lung function and the suppression of inflammatory responses (177).

Regarding inflammation-related conditions, a number of compounds have demonstrated the ability to suppress inflammation (178–180). For example, *Pistacia weinmannifolia* can significantly reduce the number of neutrophils in the bronchoalveolar lavage fluid of mice with CS- and LPS-induced lung inflammation, while decreasing the levels of inflammatory molecules such as tumor TNF-α and IL-6, as well as toxic molecules (181). Inflammation regulation is primarily achieved through NF-κB pathway inhibition. Bioactive compounds including *Astragaloside IV* (182), Baicalin (183), Morin (184), Nucleosides from *Ophiocordyceps sinensis* (185), *Luteolin* (186), *Codonopsis Radix* (187) and *C. sinensis* (188) can alleviate lung inflammation of COPD. For example, *Astragaloside IV* and Baicalin have been found to effectively reduce the levels of TNF-α, IL-6 and IL-1β. On the other hand, Morin and nucleosides extracted from *Ophiocordyceps sinensis* have significantly decreased the levels of inflammatory mediators in both the bronchoalveolar lavage fluid (BALF) and serum of mice with COPD.

In terms of oxidative stress, some compounds can improve the situation by modulating specific pathways such as Nrf2 and PERK (189–191). For example, Ursolic acid effectively alleviates CS-induced apoptosis of lung cells by downregulating the PERK pathway and significantly improves the oxidative stress response in the lungs of rats induced by cigarette smoke by upregulating the Nrf2 pathway, ultimately relieving the symptoms of CS-induced emphysema in rats (192). Cryptotanshinone can increase the activities of superoxide dismutase (SOD), catalase (CAT), and L-glutathione (GSH), and regulates the level of oxidative stress in COPD (193). Luteolin exerts a multi - pronged regulatory effect on cellular functions by reducing the expression of TRPV1 and CYP2A13 while enhancing SIRT6 expression, thereby impeding calcium ion influx and decreasing intracellular ROS levels (194). Forsythiaside increases the glutathione/glutathione disulfide (GSH/GSSG) ratio, while isoliquiritigenin reduces myeloperoxidase (MPO) activity and malondialdehyde (MDA) levels, and both, through distinct mechanisms, effectively ameliorate the oxidative stress state in mice with COPD (195, 196).

By conducting in-depth research on the regulatory mechanisms of natural products on multiple targets and pathways in chronic obstructive pulmonary disease (COPD), there is hope to reveal the mystery of their synergistic effects. At the same time, through carrying out large-scale clinical studies to verify the safety and effectiveness of natural products, it is expected to achieve the transformation from laboratory research to clinical application, providing COPD patients with safer and more effective treatment options, improving their quality of life, and reducing the disease burden.

## 5 Conclusion

COPD is a complex disease involving multiple systems. The lung and gut microbiota and the gut-lung axis of their interaction play a crucial role in its pathogenesis. Although various aspects of the prevention and treatment of COPD have been explored currently, such as targeted therapies of the gut microbiota showing certain potential and diet and natural therapies also having positive significance, many challenges still remain. Fecal microbiota transplantation has difficulties in safety monitoring and donor screening. The clinical application effect of probiotics and prebiotics requires more evidence to support. The role of vitamins in COPD is controversial and the causal relationship is not completely clear. The preventive effect of dietary fiber varies depending on the source. The in-depth research on the mechanism of natural therapies is also urgent. In the future, it is necessary to further explore the pathogenesis of COPD, optimize the existing prevention and treatment strategies, conduct more high-quality clinical trials, so as to improve the comprehensive prevention and treatment level of COPD, improve the quality of life of patients and reduce the social and economic burden.
